# Targeted Protein Degradation: Principles and Applications of the Proteasome

**DOI:** 10.3390/cells12141846

**Published:** 2023-07-13

**Authors:** Yosup Kim, Eun-Kyung Kim, Yoona Chey, Min-Jeong Song, Ho Hee Jang

**Affiliations:** 1 Department of Biochemistry, College of Medicine, Gachon University, Incheon 21999, Republic of Korea; youandkys@naver.com (Y.K.); ekkim@gachon.ac.kr (E.-K.K.); yoona.chey@gmail.com (Y.C.); neptune6nrg@hanmail.net (M.-J.S.); 2 Department of Health Sciences and Technology, Gachon Advanced Institute for Health Sciences and Technology (GAIHST), Gachon University, Incheon 21999, Republic of Korea; 3 Lee Gil Ya Cancer and Diabetes Institute, Gachon University, Incheon 21999, Republic of Korea

**Keywords:** proteasome, targeted protein degradation, E3 ubiquitin ligase, ubiquitin, PROTAC

## Abstract

The proteasome is a multi-catalytic protease complex that is involved in protein quality control via three proteolytic activities (i.e., caspase-, trypsin-, and chymotrypsin-like activities). Most cellular proteins are selectively degraded by the proteasome via ubiquitination. Moreover, the ubiquitin–proteasome system is a critical process for maintaining protein homeostasis. Here, we briefly summarize the structure of the proteasome, its regulatory mechanisms, proteins that regulate proteasome activity, and alterations to proteasome activity found in diverse diseases, chemoresistant cells, and cancer stem cells. Finally, we describe potential therapeutic modalities that use the ubiquitin–proteasome system.

## 1. Introduction

Reactive oxygen species (ROS), by-products of aerobic metabolism, are mostly produced in the mitochondria [1]. Low concentrations of ROS are involved as signaling molecules in various pathways, and high concentrations of ROS are removed by antioxidant proteins before they damage cells [2,3]. Nevertheless, excessive production of ROS causes imbalances between the production of free radicals, and their elimination and can lead to misfolding of native proteins [4]. To prevent the accumulation of misfolded proteins, cells have developed protein quality control machinery such as molecular chaperones [5]. Chaperone proteins facilitate the refolding of misfolded proteins; however, limitations exist since some proteins cannot be refolded to their native state by chaperones; examples include damaged or misfolded soluble proteins and obsolete proteins [6,7]. Moreover, when dysregulation of protein homeostasis (proteostasis) continues, it causes diseases such as cancer, amyotrophic lateral sclerosis, metabolic disorders, and spinal and bulbar muscular atrophy [8,9,10]. Therefore, the timely removal of non-native proteins is essential for cells to maintain protein homeostasis.

The main method of eliminating non-native proteins from cells is protein degradation (proteolysis). There are two main pathways involved in proteolysis in cells: the ubiquitin–proteasome system (UPS) and the autophagy–lysosome system [11]. Of these two, UPS prevents the abnormal accumulation of proteins by directly breaking down more than 80% of cellular proteins [12]. In contrast, the autophagy–lysosome system is involved in the decomposition of proteins that cannot be degraded even by the proteasome, such as insoluble or aggregated proteins, cellular organelles, etc. [13,14]. Thus, the proteasome is essentially involved in protein quality control against the accumulation of aggregated proteins.

Recently, various treatment methods such as proteolysis targeting chimera (PROTAC) and molecular glues have been developed for targeted protein degradation (TPD). Preclinical results from 2019 show distinct promise of this technique in the future [15]. In such treatment methods, the UPS is mainly involved in selectively degrading target proteins. Moreover, unlike existing treatments (i.e., small molecule inhibitors, siRNAs, and monoclonal antibodies, among others), this method is a new approach that can directly target and remove disease-causing proteins [16]. Thus, the development of treatments using the proteasome is expected to increase in the future. In this review, we briefly describe the structure of the proteasome, the mechanistic basis of the UPS, and the regulatory proteins involved in proteasome activity. Finally, we conclude by discussing the possibility of various therapeutic modalities using the proteasome.

## 2. Ubiquitin

Ubiquitin (originally called “ubiquitous immunopoietic polypeptide”) was first discovered by Gideon Goldstein in 1975 [17]. Five years after its discovery, Keith D. Wilkinson, Michael K. Urban, and Arthur L. Haas confirmed that a small heat-stable polypeptide called adenosine triphosphate (ATP)-dependent proteolysis factor1 (APF1) was actually ubiquitin [18]. In 1977, Alfred Goldberg first demonstrated ATP-dependent protein degradation in reticulocytes [19]. Three years later, Aaron Ciechanover, Avram Hershko, and Irwin A. Rose suggested that the ATP-dependent covalent binding of ubiquitin was required for targeting proteins for degradation; they were awarded the Nobel Prize in Chemistry in 2004 for the discovery of ubiquitin-mediated protein degradation. After that, extensive research on ubiquitin was steadily conducted in the following decades [20,21,22].

### 2.1. Type and Function of Ubiquitin Chains

**Figure 1:**
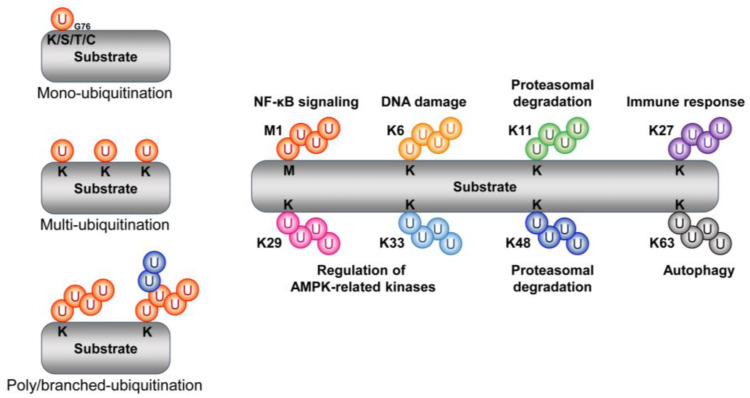
Schematic representation of the type and function of ubiquitin chains. Ubiquitin forms an isopeptide bond with substrate side-chain lysine (K), serine (S), threonine (T), and cysteine (C) residues through its C-terminal glycine residue 76 (G76). These chains, through mono-, multi-, and polyubiquitination, participate in various cellular signaling pathways. The representative sites and functions of the polyubiquitination chain are as follows (right).

Ubiquitin is a small globular protein composed of 76 amino acid polypeptides (8.6 kDa) [23]. As its name suggests, ubiquitin is ubiquitously present and is highly conserved in a wide range of eukaryotes from yeast to humans [24]. Ubiquitination (also referred to as ubiquitylation) is one of the most prevalent reversible post-translational modifications. It involves the C-terminal glycine (Gly76) of ubiquitin forming an isopeptide linkage with the internal lysine, serine, threonine, and cysteine of a target protein, followed by the subsequent formation of a mono- or polyubiquitinated chain [25,26] (Figure 1). Therefore, the complexity of the ubiquitin chain with respect to type and length constitutes its ubiquitin code [27]. Ubiquitin N-terminal methionine (i.e., Met1/linear) and seven internal lysine (K) residues (i.e., K6, K11, K27, K29, K33, K48, and K63) are involved in the formation of eight different types of ubiquitin linkages [28]. Moreover, ubiquitin is involved in diverse biological processes including protein trafficking, protein degradation, DNA repair, NF-κB activation, endocytosis, and cell cycle progression, depending on the linkage position [29,30,31].

#### 2.1.1. Methionine 1

The sequential binding of ubiquitin to methionine 1 (Met1) of ubiquitin causes the formation of linear polyubiquitination chains (i.e., Met1-linked or linear ubiquitin chains). At the same time, the linear ubiquitin chain assembly complex (LUBAC), a ubiquitin ligase complex consisting of SHARPIN, HOIP, and HOIL-1L, is involved in Met1/linear-linked ubiquitin chain formation. LUBACs are known to stimulate the NF-κB activation pathway via LUBAC-mediated linear polyubiquitin chains [32,33].

#### 2.1.2. Lysine 6 (K6)

The tumor suppressor BRCA1 is implicated in both DNA repair and cell cycle regulation. In addition, BRCA1 has E3 ubiquitin ligase activity due to the presence of a novel gene (RING) finger domain located at its N-terminus. BRCA1 forms a heterodimeric complex with its N-terminal binding partner BARD1. The E3 ubiquitin ligase activity of BRCA1 is enhanced by BARD1, resulting in the auto-ubiquitination of BRCA1. The resulting ubiquitin chain is specifically formed at the K6 residue of ubiquitin. This K6-linked ubiquitination is believed to be associated with DNA double-strand break repair [34].

#### 2.1.3. Lysine 11 (K11)

K11, together with K48, is involved in the proteasomal degradation of substrates. E3 ubiquitin ligase anaphase-promoting complex/cyclosome (APC/C), a key regulator of the cell cycle, is known to regulate mitosis and G1 phase. Substrates (e.g., cyclins) are bound to APC/C through the coactivator cell division cycle 20 (CDC20) or cadherin 1, and K11-linked ubiquitination and subsequent degradation is facilitated via E2 UbcH10 and Ube2S [35,36]. In another example, an E3 ubiquitin ligase neural precursor cell-expressed developmentally downregulated gene 4 (NEDD4) has been found to induce the K11-linked proteasomal degradation of Beclin1 [37].

#### 2.1.4. Lysine 27 (K27)

Voltage-dependent anion channel 1 (VDAC1) is a target of the E3 ubiquitin ligase Parkin and is involved in the autophagy of damaged mitochondria (“mitophagy”), during which K27-linked ubiquitin chains are generated [38]. Upon cytokine stimulation, the E3 ubiquitin ligase Itch is activated and induces the K27-linked polyubiquitination of B-Raf. After activation, B-Raf then promotes tumorigenesis in melanoma cells by activating MEK/ERK signaling [39]. Another example of a protein with K27-linked ubiquitin chains is TIEG1, which is an essential transcription factor for TGF-b-induced regulatory T cell (Treg) development. The proinflammatory cytokine IL6 activates the tyrosine (Tyr) kinase Tyk2 to phosphorylate TIEG1 at Tyr179. Phosphorylated TIEG1 then undergoes K27-linked polyubiquitination via the E3 ubiquitin ligase Itch. K27-linked polyubiquitination in turn suppresses the nuclear translocation of TIEG1, suppresses Foxp3 expression and TGF-b-induced Treg development, and increases Th17 cell development, thereby reducing tumor growth [40]. In addition, K27 is involved in the innate immune response via various target proteins (e.g., cGAS and STING) [41,42].

#### 2.1.5. Lysine 29 (K29)

AMP-activated protein kinase (AMPK)-related kinases, such as AMPK-related kinase NUAK family kinase1 (NUAK1) and microtubule-affinity-regulating kinase 4 (MARK4) are ubiquitinated by K29-linked ubiquitin chains. The polyubiquitination of NUAK1 and MARK4 was found to result in no alteration of its stability, but inhibited both its phosphorylation and activity [43]. Finally, atrophin-1-interacting protein 4 has been found to induce the polyubiquitination of deltex via K29-linked ubiquitin chains for lysosomal degradation [44].

#### 2.1.6. Lysine 33 (K33)

The E3 ubiquitin ligase Parkin is phosphorylated and activated by AMPK. Activated Parkin then inhibits the pronecroptotic factor RIPK1−RIPK3 interaction by promoting the polyubiquitination of RIPK3 via K33-linked ubiquitin chains (note that the protein level of RIPK3 is not changed by Parkin). Through this pathway, the AMPK–Parkin axis inhibits necroptosis [45].

#### 2.1.7. Lysine 48 (K48)

K48-linked ubiquitin chains are the most intensively studied ubiquitin modification related to proteasome-mediated protein degradation. Most proteins, including p53, HIF1α, HIPK2, and ASK1, are ubiquitinated via the ubiquitin K48, and K48-linked ubiquitin chains act as a degradation signal to induce the proteasomal degradation of proteins [46,47,48,49,50]. Table 1 summarizes the proteins degraded via the 20S proteasome and those degraded via the ubiquitin-dependent or -independent 26S proteasome (Table 1).

#### 2.1.8. Lysine 63 (K63)

The E3 ubiquitin ligase TRAF6 promotes the ubiquitination of hexokinase 2 (HK2), a glycolytic enzyme that converts glucose to glucose-6-phosphate, at the K41 residue using K63-linked ubiquitin chains. Under high autophagic flux, ubiquitinated HK2 is recognized by the autophagy receptor SQSTM1, also known as p62, leading to the formation of autophagosomes and the subsequent selective autophagic degradation, thereby suppressing glycolysis. However, under conditions of low autophagic flux, the formation of autophagosomes is impaired, resulting in the absence of HK2 degradation. These findings demonstrate that autophagy modulates HK2-dependent glycolysis [82].

## 3. The Proteasome and Beyond

The proteasome participates in direct protein degradation as a high-molecular-weight protease complex. The two major species of proteasome are the 26S proteasome (2.5 MDa) and the 20S proteasome (700 kDa) [83]. The 26S proteasome consists of the 20S proteasome (also called 20S core particle, CP) and one or two 19S regulatory particles (RPs, also called PA700). The 20S proteasome is composed of two of outer heptameric rings, each containing seven α-subunits (α1–α7), and two inner heptameric rings, each containing seven β-subunits (β1–β7). These are then assembled as a β-ring barrel-shaped structure (α7-β7-β7-α7). The α-rings serve as a “gate” for substrate entry, while three subunits (i.e., β1, β2, and β5) of the β-rings hold distinct peptidase activities and directly degrade proteins [84]. The 26S proteasome engages in ATP, ubiquitin-dependent protein degradation, whereas the 20S proteasome engages in ATP, ubiquitin-independent protein degradation [85]. In addition, the type and the number of regulatory particles that interact with the 20S proteasome accounts for the diversity of proteasome complexes [86,87].

### 3.1. Assembly of the 20S Proteasome

**Figure 2:**
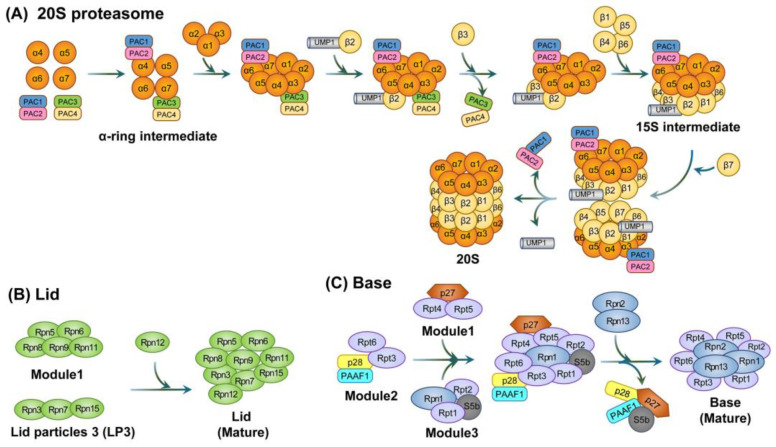
The assembly of the 20S proteasome and two parts of the 19S regulatory particle, the lid and base subcomplexes. (**A**) Schematic diagram of the 20S proteasome. For synthesis of the 20S proteasome, the α-ring is formed first, followed by the formation of the β-subunit, thereby forming the β ring. Of the β-subunits, the catalytically active subunits that directly degrade are β1, β2, and β5. (**B**) Schematic diagram of the lid subcomplex. Rpn12 is an important subunit that binds to LP2 (i.e., module1 + LP3) to form the lid subcomplex. This is involved in base binding via Rpn10. (**C**) Schematic diagram of the base subcomplex. The basic subcomplex directly linked to 20S proteasome is complexed by various chaperone proteins. The lid subcomplex and base subcomplex are collectively referred to as 19S RPs.

For the 20S proteasome assembly, a proteasome-assembling chaperone 1 (PAC1)-PAC2 hetero-dimer and a PAC3-PAC4 hetero-dimer first create α-ring intermediates from the α4, α5, α6, and α7 structures within α-subunits. PAC1-PAC2 and PAC3-PAC4 prevent the off-pathway dimerization of α-subunits and the incorrect incorporation of the other α-ring, thereby facilitating stable α-ring formation. Once the α1, α2, and α3 subunits are assembled into an α-ring, the β-subunit starts to be incorporated. The β2 subunit then associates with the α-ring, which is promoted by ubiquitin-mediated proteolysis 1 (UMP1), and the subsequent binding of β3 dissociates the PAC3-PAC4 complex. Next, the remaining β-subunits (but not β7) are incorporated to form the 15S intermediate complex. Subsequent β7 incorporation into the 15S intermediate creates a complex, termed the ‘half-proteasome’, that triggers its own dimerization. Moreover, UMP1 is required for proper maturation of the 20S proteasome since it inhibits the premature dimerization of half-proteasomes. Finally, the mature 20S proteasome degrades the assembly factors such as UMP1 and the PAC1-PAC2 hetero-dimer (in yeast, PAC1-PAC2 is recycled for future assembly into new 20S proteasomes) (Figure 2) [88,89,90].

### 3.2. Assembly of the 19S RP

The 19S RP can be subdivided into the lid subcomplex (~370 kDa) and the base subcomplex [91,92]. The lid subcomplex consists of nine regulatory particle non-ATPase (Rpn) subunits (i.e., Rpn3, Rpn5, Rpn6, Rpn7, Rpn8, Rpn9, Rpn11, Rpn12, and Rpn15). The base subcomplex consists of six regulatory particle ATPase (Rpt) subunits (i.e., Rpt1, Rpt2, Rpt3, Rpt4, Rpt5, and Rpt6) as well as three non-ATPase subunits (i.e., Rpn1, Rpn2, and Rpn13) [93]. Rpn10, which was thought to be a part of the base subcomplex, is now considered to be a factor that stabilizes the lid–base interaction and recognizes the ubiquitin chain [94,95].

The lid subcomplex assembly begins as two subcomplexes, module 1 (consisting of Rpn5, Rpn6, Rpn8, Rpn9, and Rpn11) and lid particle 3 (LP3; consisting of Rpn3, Rpn7, and Rpn15), which join to form lid intermediate LP2. Rpn12 is then incorporated into the LP2 intermediate to complete the lid subcomplex [92,96].

**Figure 3:**
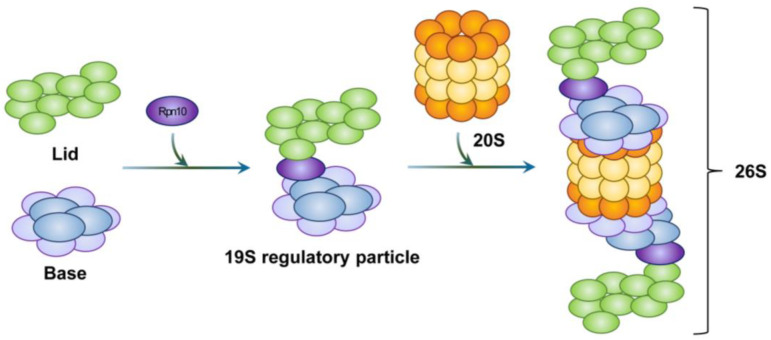
Schematic representation of proteasome structure. Rpn10 acts as a hinge between the 20S proteasome and the 19S regulatory particle. Rpn10 is also involved in the formation of the 26S proteasome. As a result, one or two 19S regulatory particles associate with the 20S proteasome to form the 26S proteasome.

The base subcomplex assembly is preceded by the formation of intermediate complexes. These include a heterohexameric ATPase ring that contains three modules, i.e., module1 (p27, Rpt4, and Rpt5), module2 (p28, Rpt3, Rpt6, and proteasomal ATPase-associated factor1 (PAAF1)), and module3 (S5b, Rpt1, Rpt2, and Rpn1). This formation is regulated by four RP assembling chaperones (RACs). These include p27, p28, S5b, and PAAF1 [97]. Subsequent incorporation of the Rpn2-Rpn13 hetero-dimer causes the dissociation of RACs. Moreover, the binding of the stabilizing factor Rpn10 to the lid and base subcomplexes completes the proper assembly of the 19S RP (Figure 3). For regulatory particles, in addition to 19S RP, various regulatory particles such as 11S RP (also called proteasome activator 28, PA28, or REG), PA200 (also known as Blm10 in yeast), ATPases associated with diver cellular activities (AAA+) ATPase forming ring-shaped complexes (ARC), a homologue of 19S RP in eubacteria, and proteasome-activated nucleotidase (PAN), a homologue of 19S RP in archaea, have been identified.

### 3.3. Assembly of the 26S Proteasome

The extracellular matrix 29 (Ecm29) proteins stabilize the 26S proteasome by tethering the 20S proteasome and the 19S RP [88,98]. Furthermore, under oxidative stress conditions, ECM29 accelerates the disassembly of the 26S proteasome [99]. Furthermore, heat shock protein 90 (Hsp90) also contributes to the association of the 26S proteasome, both in vivo and in vitro, in an ATP-dependent manner [100].

### 3.4. Steps Involved in Substrate Degradation via the 26S Proteasome

#### 3.4.1. Ubiquitination

**Figure 4:**
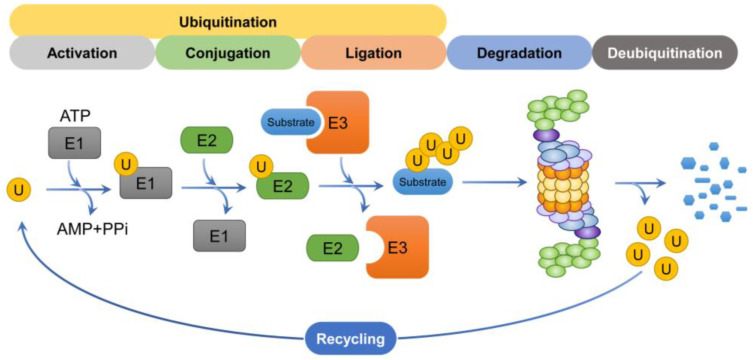
Schematic representation of the degradation process. The ubiquitin mediates the K48-linked ubiquitination of substrates through a cascade involving E1, E2, and E3 enzymes. Ubiquitin chains are recognized by the 19S regulatory particle and subsequently degraded by the 26S proteasome. The ubiquitin molecules that participated in the degradation process are then recycled to participate in substrate degradation again.

Aberrant proteins, including damaged proteins, misfolded proteins, over-expressed protein substrates, and short-lived proteins, require ATP-dependent proteolysis to be degraded by the 26S proteasome. (For some proteins, which are degraded by the 20S proteasome via an ubiquitin-independent pathway, a sequential cascade for protein degradation called ubiquitination is not required) [101] (Figure 4). Of the various types of ubiquitin chain mentioned above, the K48-linked polyubiquitin chain, which is involved in proteasomal degradation, is the most abundant and widely studied [102]. The process of ubiquitination requires three different enzymes: E1 ubiquitin-activating enzymes (of which there are 2), E2 ubiquitin conjugases (~40 in total), and E3 ubiquitin ligases (over 600 in total) [103]. These are involved in the sequential ubiquitination process as follows: Step 1: ubiquitin is activated when a thioester bond forms between the C-terminal glycine (i.e., Gly76) of ubiquitin and the active site cysteine of the E1 ubiquitin-activating enzyme in an ATP-dependent manner. Step 2: activated ubiquitin is transferred from the E1 ubiquitin-activating enzyme to E2 ubiquitin conjugase, and a thioester bond forms between the C-terminal glycine of ubiquitin and the active site cysteine of the E2 ubiquitin conjugase. Step 3: when the ubiquitin–E2 complex and substrate bind to E3 ubiquitin ligase, E3 ubiquitin ligase transfers ubiquitin to the substrate. Consequently, a covalent peptide bond is formed between the C-terminal Gly of ubiquitin and a lysine residue in the substrate protein. Step 4: in some cases, an E4 ubiquitin ligase, a polyubiquitin chain elongation factor, is involved in polyubiquitin chain assembly [104,105,106].

The substrate specificity of E3 ubiquitin ligase is a significant feature involved in the ubiquitination process. E3 ubiquitin ligases are subdivided into four groups, including: (1) the RING-type E3 ubiquitin ligases; (2) the homology to E6-associated protein carboxyl terminus (HECT)-type E3 ubiquitin ligases; (3) the RING-between-RING (RBR) E3 ubiquitin ligases, also referred to as RING-HECT hybrids; and (4) the U-box-type E3 ubiquitin ligases [107,108]. RING-type E3 ubiquitin ligases have a RING domain and a substrate-binding domain (SBD). When ubiquitin-E2 ubiquitin conjugase binds to the RING domain of E3 ubiquitin ligases, the ubiquitin on the E2 ubiquitin conjugase is directly transferred to the substrate bound to the SBD, whereas ubiquitination via HECT type E3 ubiquitin ligases requires an additional step in which the ubiquitin is first bound to a catalytic cysteine (Cys) residue in the HECT domain within a HECT type E3 ubiquitin ligase. Here, the ubiquitin is subsequently transferred to the substrate. RBR-type E3 ubiquitin ligases have two RING domains. Once ubiquitin-E2 ubiquitin conjugase binds the RING1 domain, the ubiquitin on the E2 ubiquitin conjugase on the RING1 domain is transferred to a catalytic cysteine of the RING2 domain and is then transferred again to the substrate. U-box type E3 ubiquitin ligases were first identified in the yeast *Saccharomyces cerevisiae* (*S. cerevisiae*) [109,110]. These ligases have a conserved U-box domain that is similar to the RING domain. In these ligases, E2 ubiquitin conjugase interacts with the U-box domain and subsequently promotes the ubiquitination of the substrate. E4 ubiquitin ligase E4B, a mammalian homologue of yeast UFD2, is a *U*-*box*-containing protein that has been found to promote the elongation of polyubiquitin chains like E3 ubiquitin ligase [111,112].

#### 3.4.2. Recognition of Ubiquitin Chains

The ubiquitin chain is recognized by three ubiquitin receptors, Rpn1, Rpn10, and Rpn13, each of which is a component of a proteasome subunit [113,114]. Rpn10 and Rpn13 are the major ubiquitin-binding subunits. In both cases, ubiquitin binds to Rpn10 and Rpn13 via a specific ubiquitin-interacting motif also known as a LALAL motif domain and a pleckstrin-like receptor for ubiquitin domain, respectively [94,115]. In addition, Rpn13 is the subunit to which the deubiquitinating enzyme UCHL5 binds [116]. Moreover, Rpn1 binds to ubiquitin through its proteasome/cyclosome repeats [117]. Rpn1 is a ubiquitin receptor and a subunit bound by the deubiquitinating enzyme USP14 [118,119].

#### 3.4.3. Deubiquitination

Deubiquitinating enzymes (DUBs) are proteases that regulate the ubiquitin–proteasome pathway. They do so by processing ubiquitin precursors into mature ubiquitin, upon which the ubiquitin molecules are cleaved from ubiquitin-conjugated substrates. This process is therefore termed deubiquitination, since DUBs prevent protein degradation by reversing the ubiquitin process. While ubiquitinated substrates are degraded by proteasomes, DUBs prevent the degradation of substrates by separating them from substrates to recycle ubiquitin monomers for the subsequent degradation of other substrates. Human DUBs are classified into two classes: cysteine proteases and metalloproteases [120]. Cysteine proteases make up most DUB members in eukaryotic cells and can be divided into seven families: (i) ubiquitin-specific proteases, (ii) ovarian tumor proteases, (iii) monocyte chemotactic protein-induced proteases, (iv) ubiquitin C-terminal hydrolases proteases, (v) Machado–Joseph domain proteases, (vi) motif interacting with Ub-containing novel DUB family, and (vii) zinc finger (ZnF) with UFM1-specific peptidase domain protein. Interestingly, the metalloproteases have an additional family: (viii) Jab1/Pab1/MPN domain-containing metalloenzymes [121,122].

The catalytic triad of cysteine proteases is mainly composed of conserved cysteine (Cys, C), aspartate (Asp, D), and histidine (His, H) residues. These proteases hydrolyze the isopeptide bond between ubiquitin and a substrate, or between ubiquitin moieties [123]. Two DUBs (i.e., USP14 and UCHL5) of the three proteasome-associated DUBs belong to the cysteine protease family and therefore cleave the ubiquitin–ubiquitin interaction once the proteasome binds to the substrate, thereby preventing degradation by the proteasome. In contrast, Rpn11, a subunit consisting of the lid subcomplex of 26S proteasome, belongs to the metalloprotease family, and is activated by the coordination between four conserved residues (i.e., His, Asp, Glutamate, and serine) and zinc ions to catalyze isopeptide hydrolysis [120,124,125]. Once the ubiquitin chain of the substrate is recognized by Rpn1, Rpn10, and Rpn13, Rpn11—unlike USP14 and UCHL5—releases whole ubiquitin from a partially unfolded substrate before it is degraded by the proteasome [126,127]. Finally, the substrate is unfolded and translocated into the 20S proteasome.

#### 3.4.4. Gate Opening and Translocation

The AAA+ enzymes are a superfamily of proteins and use the energy produced when ATP is hydrolyzed to ADP to drive various cellular activities [128]. Ring-shaped heterohexameric AAA+ complexes, which consist of the six ATPases (Rpt1, Rpt2, Rpt3, Rpt4, Rpt5, and Rpt6), mechanistically unfold and translocate substrates into the 20S proteasome. Rpt2, Rpt3, and Rpt5 also contain a conserved C-terminal hydrophobic-tyrosine-X (HbYX) motif, which interacts with the lysine-pocket (K-pocket) of the α-ring of the 20S proteasome. This triggers the gate opening of the α-ring pore of the 20S proteasome to convert it from a closed to an open state after ATP binding. Six ATPases change their confirmation to adopt a spiral staircase arrangement in an ATP-dependent manner, thereby permitting substrate unfolding and translocation into the 20S proteasome [129,130].

#### 3.4.5. Proteolysis

Unfolded substrates translocated into the proteolytic chamber via the α-ring are then directly degraded by β-subunits. Of the seven β-subunits, the β3, β4, β6, and β7 subunits participate in the assembly of the structural complex of the 20S proteasome, whereas β1, β2, and β5 display caspase-like (or peptidylglutamyl-peptide-hydrolyzing), trypsin-like, and chymotrypsin-like activities, respectively [131]. β1 hydrolyzes the peptide bond on the carboxyl side of acidic or hydrophobic amino acids. Moreover, β2 cleaves the bond on the carboxyl side of basic or hydrophobic amino acids, and β5 cleaves the bond on the carboxyl side of hydrophobic amino acids. The cylindrical particle of 20S proteasomes contains two β-rings and is responsible for six substrate-degrading activities. In addition, 20S proteasomes contain highly conserved threonine (Thr) residues on the N-termini of all active β-subunits and is therefore called a Thr protease [132]. Moreover, given the proteolytic activities within the cylindrical core particle, the isopeptide bonds of the substrate are hydrolyzed, resulting in the generation of small peptides. Their average length ranges between 3 and 23 amino acids [84,85,133].

### 3.5. Substrate Degradation via the 20S Proteasome

In mammalian cells, approximately 20% of the proteasomes are 26S proteasomes, while the rest consist of 20S proteasomes, immunoproteasomes, thymoproteasomes, and hybrid proteasomes [134]. Despite this distribution, around 80% of the total protein is degraded by the 26S proteasome [12]. As mentioned before, the 26S proteasome recognizes substrates linked to ubiquitin chains, which are typically associated with natively folded proteins, for degradation. In contrast, the 20S proteasome is known for its role in the default degradation of proteins that exhibit certain characteristics. These include proteins containing intrinsically disordered regions (IDRs) or being intrinsically disordered proteins (IDPs) [135]. Some proteins exist in a partially or fully unfolded state without forming a defined structure. Proteins with such disordered regions, such as p21, p53, c-fos, α-synuclein, tau, and others, are degraded by the ubiquitin-independent 20S proteasome [136]. Additionally, the 20S proteasome is involved in the degradation of oxidatively damaged proteins [137]. Oxidative stress induces the dissociation of the 26S proteasome into the 20S proteasome and the 19S RP, leading to the accumulation of structurally unstable misfolded proteins. Misfolded proteins, exposing hydrophobic regions, tend to aggregate. The 20S proteasome recognizes these hydrophobic regions and facilitates the degradation of oxidized proteins [138]. However, it has recently been reported that ubiquitin-tagged proteins can also be degraded through the 20S proteasome [139]. In addition, proteins such as p53 and p21 have been shown to undergo degradation by both the 26S proteasome and the 20S proteasome [140]. This indicates that the degradation mechanisms mediated by the 26S proteasome and the 20S proteasome are not mutually exclusive but rather complementary to each other [136].

### 3.6. Mixed Proteasome

The mixed proteasomes, also known as intermediate proteasomes, contain a combination of immune and constitutive proteolytic subunits: β1, β2, and β5i (also known as LMP7, with chymotrypsin-like activity); β1i (also known as LMP2, with chymotrypsin-like or branched-chain-amino-acid-preferring activity), β2, and β5i. These proteasome subtypes expand the repertoire of antigens presented to CD8+ T cells [141]. In addition, proteasome subtype β1i, β2i (also known as MECL-1, with trypsin-like activity), and β5, without β5i, can be formed [142]. The high expression of proteasomes containing subunit β1i, but not β5i, is associated with the development of immunological tolerance [143]. β1i is required for the adaptation of rat ventricular cardiomyocytes to reach pressure overload [144]. Thus, the functions of proteasomes containing immune subunits in combination with constitutive ones are wider than the formation of antigenic epitopes.

### 3.7. Immunoproteasome

The immunoproteasome is an alternative form of the constitutive 20S proteasome that was discovered in 1994 [145]. It has distinct catalytic subunits known as β1i, β2i, and β5i. In contrast to the regular proteasome, the immunoproteasome contains these specialized subunits, which confer unique enzymatic activities. The synthesis of these subunits, β1i, β2i, and β5i, is induced by pro-inflammatory cytokines such as tumor necrosis factor-alpha (TNF-α), interferon-gamma (IFN-γ), and oxidative stress. Consequently, the immunoproteasome is generated ahead of the constitutive 20S proteasome [86,146]. Immunoproteasomes are known to be highly expressed in immune cells, such as antigen-presenting cells (APCs). They play a crucial role in generating antigenic peptides by breaking down intracellular antigens. These antigenic peptides are then transported into the endoplasmic reticulum (ER), where they form complexes with newly synthesized major histocompatibility complex (MHC) class I molecules. Subsequently, these complexes undergo processing in the Golgi apparatus and are presented on the cell surface. Once presented on the cell surface, the antigenic peptide–MHC complex can be recognized by the T cell receptors (TCRs) of CD8+ T cells, leading to the triggering of immune responses [147,148].

### 3.8. Thymoproteasome

The thymoproteasome, discovered in 2007, is closely related to the positive selection of T cells [149]. It is exclusively expressed in cortical thymic epithelial cells (cTECs) within the cortex and shares structural similarities with the immunoproteasome, containing unique subunits called β1i, β2i, and β5t [150]. The maturation of T cells occurs as immature T cells, formed in the bone marrow, which migrate to the thymus. The thymus can be divided into the cortex and medulla, and it is in the cortex where the initial development of T cells takes place through positive or negative selection, ultimately leading to the formation of mature T cells. Within cTECs, self-antigens are degraded by the thymoproteasome into self-peptides. These self-peptides, along with MHC class I molecules, are formed in the ER and presented on the surface of cTECs after passing through the Golgi apparatus. The T TCRs of T cells recognize these self-peptides, determining the outcome of positive selection. T cells with weak binding to self-peptides undergo positive selection, differentiating into mature CD8+ T cells that participate in adaptive immune responses. On the other hand, T cells with strong binding to self-peptides undergo negative selection, leading to apoptosis. This process prevents the development of T cells that could potentially cause autoimmune diseases [146,151].

## 4. Proteasome Regulatory Proteins

### 4.1. Proteasome-Activating Proteins

#### 4.1.1. 11S Regulatory Particle

The 11S RP was first identified in bovine and human red blood cells in 1992 [152,153]. Due to its molecular weight of approximately 28 kDa, it is also known as proteasome activator 28 (PA28) or REG. In mammals, three homologous subunits of 11S RP (PA28α, PA28β, and PA28γ) have been identified. Among them, PA28α (28.7 kDa) and PA28β (27.1 kDa) subunits share about 47% sequence identity. PA28α and PA28β form heteroheptameric ring structures, predominantly in the form of α4β3 and α3β4 complexes [154,155]. They are primarily localized in the cytosol. On the other hand, PA28γ forms homoheptamers and is mainly localized in the nucleus [156]. 11S RP increases all three activities of the 20S proteasome, but there is no difference in the degradation rate compared to when only the 20S proteasome is present. Additionally, 11S RP facilitates the degradation of small peptides, but it is unable to degrade large proteins and ubiquitin-conjugated proteins [157,158]. Finally, 11S RP is induced by IFN-γ and binds to 20S proteasome in an ATP-independent manner, resulting in the formation of either 11S-20S proteasome-11S or 11S-20S proteasome-19S hybrid proteasomes (Table 2) [159].

#### 4.1.2. PA200

PA200, another proteasome activator, is primarily localized in the nucleus and has a molecular weight of 200 kDa [160]. It binds to the 20S proteasome alone or in conjunction with the 19S RP, forming a hybrid proteasome complex (PA200-20S proteasome-19S RP), thereby enhancing the peptidase activity of the 20S proteasome [161]. PA200 stimulates the hydrolysis of small peptides and unstructured proteins, such as tau, in an ATP-dependent manner [162]. Additionally, it is involved in DNA repair and the degradation of acetylated histones [160,163].

#### 4.1.3. Nuclear Respiratory Factor 1

Nuclear respiratory factor 1 (NRF1), also known as the endoplasmic reticulum membrane protein, is retrotranslocated to the cytosol by p97 under normal conditions and is degraded by the 26S proteasome via an ER-associated degradation pathway. However, when proteasome activity is impaired or insufficient, NRF1 is cleaved, releasing a soluble 100 kDa fragment (i.e., p110, an active form of NRF1) via aspartic protease DNA Damage Inducible 1 Homolog 2 (DDI2) to the cytosol. p110 is then translocated to the nucleus, where it dimerizes with the cofactor small musculoaponeurotic fibrosarcoma to induce proteasome gene expression, thereby recovering proteasome activity [164,165].

#### 4.1.4. Zinc Finger AN1-Type Containing 5

Zinc finger AN1-type containing 5 (ZFAND5) is a protein that has been found to increase muscle atrophy. It directly binds to the 20S proteasome and stimulates three peptidase activities of the 26S proteasome. In particular, the AN1 domain near the C-terminus of ZFAND5 is essential for the stimulation of peptidase activity. ZFAND5 also enhances the hydrolysis of ubiquitinated dihydrofolate reductase (DHFR) by increasing ATPase activity. As a result, ZFAND5 promotes total protein degradation via the ATP- and ubiquitin-dependent 26S proteasome pathway [166].

#### 4.1.5. Tankyrase

The 20S proteasome displays low activity when bound to a proteasome inhibitor of 31 kDa (PI31). In addition, ADP-ribotransferase tankyrase (TNKS) mediates ADP-ribosylation of PI31, which then combines with the 19S RP assembly chaperones p27 and S5b (which was previously bound to 19S RP), and the dissociated 19S RP then binds to the 20S proteasome, thereby increasing 26S proteasomal activity [167].

### 4.2. Proteasome-Inhibiting Proteins

#### 4.2.1. DJ-1

DJ-1, also known as Parkinson’s disease protein 7 (PARK7), is a multifunctional protein associated with Parkinson’s disease, cancer, oxidative stress response, and mitophagy. DJ-1 binds to the 20S proteasome and inhibits its activity. Through this mechanism, α-synuclein and p53, substrates known to be degraded through the 20S proteasome, were instead protected [168].

#### 4.2.2. NAD(P)H:Quinone-Oxidoreductase 1

NQO1 is a flavin adenine dinucleotide (FAD)-dependent flavoprotein that catalyzes the reduction of quinone to hydroquinone. NQO1, a superoxide reductase, possesses an innate antioxidant activity and can directly scavenge superoxide. Furthermore, NQO1 inhibits the degradation of p53, ornithine decarboxylase (ODC), and α-synuclein by binding to the 20Sproteasome, where it acts as a gate keeper [169,170]. Apo-NQO1, in its FAD-free form, is unstable due to a structural change caused by a partially unfolded conformation; this makes Apo-NQO1 susceptible to degradation by the 20S proteasome. Therefore, NQO1 and the 20S proteasome mutually regulate their activities via a double negative feedback loop [171].

#### 4.2.3. PI31

PI31 is a proline-rich protein whose carboxyl-terminal proline-rich domain plays a role in inhibiting the activity of the 20S proteasome. PI31 competes with the proteasome regulatory proteins 19S RP and 11S RP when binding to the 20S proteasome, thereby blocking the activation of the 20S proteasome [172,173].

#### 4.2.4. c-Abl

The non-receptor Tyr kinase c-Abl binds to the PSMA7 (i.e., at its α4 subunit) of the 20S proteasome and phosphorylates Tyr residue (Y106). PSMA7 phosphorylation at Y106 inhibits the ubiquitin-dependent proteasomal degradation of PSMA7 and increases its expression. This in turn increases proteasome abundance. However, proteasome activity remains suppressed, which contrasts with the cellular proteasome levels [174,175].

#### 4.2.5. Bassoon

The presynaptic cytomatrix protein BSN is localized to the active zone of the presynaptic terminal where it interacts with the PSMB4 (i.e., at its β7 subunit) of the 20S proteasome. BSN suppresses proteasome assembly by binding to half-proteasomes at two independent regions. The reduced activity of the proteasome then leads to the inhibition of ubiquitination-dependent and independent proteolysis. Consequently, BSN reduces the degradation of presynaptic scaffolding proteins such as Rab3-interacting molecules (RIMs) and mammalian homolog of *Caenorhabditis elegans* unc-13 (Munc13), thus causing the accumulation of misfolded proteins [176].

## 5. Proteasome Activity in Diseases

### 5.1. Neurodegenerative Disease

A change of proteasomal activity was one of age-related dysfunction [177]. Especially, accumulation of misfolded proteins via alternative proteasome activity was induced in several neurodegenerative diseases, such as Alzheimer’s, Parkinson’s, and Huntington’s disease [178]. Proteasome activity was decreased in these diseases and leaded to the aggregation of ꞵ-amyloid, tau tangles, lewy bodies, and poly-glutamine inclusions [179]. Therefore, neurodegenerative disease observed neuronal loss and dysfunction [178].

### 5.2. Muscle Atrophy and Cachexia

Enhanced proteasome activity changes in the life time of a protein [180]. Muscle atrophy was observed to decrease protein synthesis; however, it was observed to increase protein degradation [178]. Cancer cachexia is also a muscle-loss-related disease [181]. Increased proteasome activity was induced in muscle protein wasting and inflammation was induced in muscle atrophy and cancer cachexia [182].

### 5.3. Chemoresistant Cells

A cisplatin-resistant neuroblastoma cell line has been found to show higher proteasome activity than a parental cell line [183]. Cisplatin-chemoresistant neuroblastoma cells have been found to show high expression levels of SHFM1 (i.e., 26S proteasome complex subunit SEM1) and PSMD14 (i.e., 26S proteasome non-ATPase regulatory subunit 14) using transcriptomic profiling [184]. In addition, the irreversible proteasome inhibitor TIR-199 effectively reduced the cell viability of bortezomib-related chemoresistance. This was found to suppress tumor growth in blood cancers such as multiple myeloma and mantle cell lymphoma [185].

### 5.4. Cancer Stem Cells

Cancer stem cells (CSCs) are characteristic of chemoresistance and tumor recurrence [186,187]. Many studies have shown that many cancer cells show high proteasomal activity. However, other studies of CSCs have reported that proteasomal activity is low [188,189]. Differences in proteasome activity have also been found among various cancers, including lung, prostate, and pancreas [189,190,191]. Moreover, the existence of CSCs has caused some solid tumors to show low proteasome activity [188]. This is notable, since low proteasome activity cells (LPACs) show increased resistance to chemotherapy and radiotherapy [187,188,192].

## 6. Therapeutic Modalities Targeting Proteasomes

**Figure 5:**
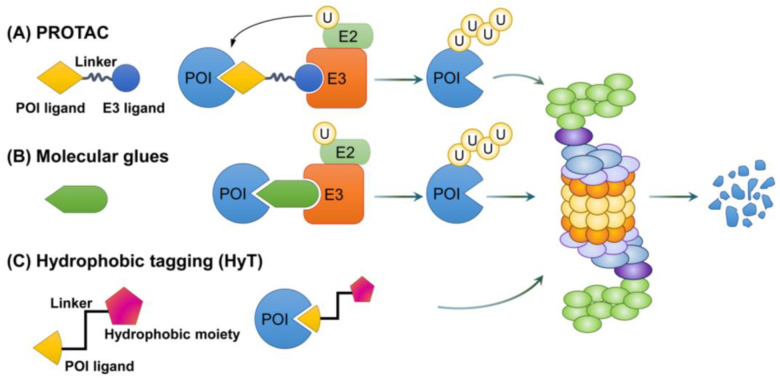
Mechanisms of action of PROTAC, molecular glues, and hydrophobic tagging. A variety of degraders are being developed for TPD. (**A**) PROTAC involves a protein of interest (POI) ligand and an E3 ligand that are linked by a linker. When POI and E3 ubiquitin ligase bind to PROTAC, the ubiquitin of E2 is sequentially attached to POI, thereby inducing ubiquitination. Ubiquitinated POIs are eventually degraded by the proteasome. (**B**) Molecular glues use a mechanism similar to PROTAC. They induce molecular proximity between a POI and E3 ubiquitin ligase, resulting in the sequential attachment of ubiquitin to the POI, which triggers its ubiquitination and degradation. (**C**) Hydrophobic tagging combines POIs to mimic hydrophobic and partially disordered forms of POIs. Recognition of chaperones at these sites then leads to chaperone-mediated proteasomal degradation.

In recent years, therapeutic modalities using TPD via the proteasome have been proposed (Table 3). These include novel strategies such as PROTAC, SNIPERs, molecular glues, and hydrophobic taggings (HyTs), and have been designed for the selective targeting of proteins associated with cancer, neurodegenerative disease, autoimmune disease, and metabolic disorders (Figure 5) [193,194].

Therefore, a TPD-using proteasome may offer the potential to target undruggable proteins and overcome disadvantages associated with conventional small molecule inhibitor-, antibody-, or gene-based therapies (i.e., those involving small molecule inhibitors, monoclonal antibodies, or RNAi), which include: broad or smooth active sites of target proteins, low tissue penetration, high molecular weight, and low oral bioavailability (Table 4). These novel therapeutic modalities are currently under active research and are being subjected to clinical trials [15,195,196].

### 6.1. Proteolysis-Targeting Chimera

Two decades ago, various pharmaceutical companies developed a modality called PROTAC technology. Currently, it is being developed as a protein degradation tool targeting various diseases. PROTAC involves a hetero-bifunctional small molecule in which a ligand of a POI and a ligand for E3 ubiquitin ligase-binding are typically linked by a linker. When POI and E3 ubiquitin ligase bind to each ligand, a ternary complex is formed. The POI is then ubiquitinated by the E3 ubiquitin ligase, thereby causing the POI to be directly degraded by the proteasome [15].

The first version of PROTAC was developed in 2001. In this system, which used a peptide-based PROTAC, the POI ligand was linked to the angiogenesis inhibitor ovalicin, which forms a covalent bond with methionine aminopeptidase2 (METAP2). As the E3 ligase-binding ligand, the IκBα peptide (DRHDSGLDSM) was used to target the E3 ubiquitin ligase, β-transducin repeat-containing E3 ubiquitin–protein ligase (β-TRCP). However, this peptide-based PROTAC showed problems related to low cell permeability, lipophilicity, and stability [197]. To solve these problems, small molecule-based, nucleotide-based, antibody-based, nanoparticle-based, and peptide-based PROTACs are currently being developed [198,199,200].

PROTAC therapies require the consideration of several points prior to development. Currently, most PROTACs bind to the E3 ubiquitin ligase component cereblon (CRBN) using immunomodulatory drugs (IMiDs) such as thalidomide, pomalidomide (Pom), and lenalidomide, which are used as E3 ubiquitin ligase binders. When CRBN binds to IMiDs, it induces POI ubiquitination by forming a complex (CRL4^CRBN^) with Cullin4-RING E3 ubiquitin ligase (CRL4), an E3 ubiquitin ligase that uses CRBN as a substrate receptor [201,202]. However, since some cells or tissues show diverse CRBN expression, new PROTAC methods are being developed that replace CRBN with other E3 ubiquitin ligases such as tumor suppressor von Hippel–Lindau, mouse double minute 2 (MDM2), and inhibitors of apoptosis (IAP) [15,203,204,205]. Moreover, when considering the ligand for the E3 ubiquitin ligase, it must be capable of high target binding specificity. Otherwise, PROTAC can generate off-target effects that affect proteins other than the POI. The POI ligand must also show a low binding affinity for the POI, since degradation via the proteasome does not occur unless the ubiquitinated POI is cleaved from PROTAC [206,207]. Furthermore, the appropriate concentration of PROTAC is also very important. PROTAC at an appropriate concentration induces the degradation of POI by forming a ternary complex structure such as POI-PROTAC-E3 ligase. However, a high concentration of PROTAC can form a binary complex as POI-PROTAC or E3 ligase-PROTAC. This phenomenon is called the “hook effect”, and results in a reduction in PROTAC activity [200]. In addition, the size of the PROTAC must also be considered. Finally, it is important to develop new PROTAC strategies, including the in-cell click-formed proteolysis-targeting chimera, which increases cell permeability by reducing molecular weight [208].

In 2019, Arvinas, Inc. conducted a Phase I trial using a PROTAC (ARV-110, NCT03888612) targeting an androgen receptor and a PROTAC (ARV-471, NCT04072952) targeting an estrogen receptor. They effectively inhibited metastatic castration-resistant prostate cancer and breast cancer, respectively, and are currently in Phase II trials. This result confirmed that PROTAC has potential as a therapeutic target. In addition, other multinational companies (e.g., Accutar Biotech, Kymera, Nurix Therapeutics, and C4 Therapeutics Inc., among others) are conducting or are planning clinical trials for various diseases using various PROTAC protocols [15].

In addition, specific non-genetic IAP-based protein eraser is another degrader molecule that works in a manner similar to that of PROTACs. This molecule features a link between a POI ligand and an antagonist (i.e., an LCL161 derivative) capable of recruiting the IAP in the presence of E3 ubiquitin ligases [209,210].

### 6.2. Molecular Glues

Molecular glues stabilize protein–protein interactions between proteins in homo- or hetero-dimer forms [211]. The most common molecular glues are small molecule degraders that mediate proximity-induced TPD. The simultaneous binding of E3 ubiquitin ligase and POI to molecular glues induces dimerization between E3 ubiquitin ligase and the POI. As a result, a ternary complex comprising molecular glue, E3 ubiquitin ligase, and the POI is formed, thereby leading to subsequent ubiquitination and proteasome-mediated degradation of the POI. The first molecular glues were serendipitously discovered. However, new molecular glues are currently under development via structure-based design, scalable chemical profiling, or microarray-based high-throughput screening. The most widely used molecular glues are the CRBN ligand thalidomide and its analogs, such as lenalidomide and Pom, and DCAF15 ligand sulfonamides. Both molecular glues and PROTAC induce the proteasomal degradation of a target protein; however, unlike PROTAC, molecular glues do not require a linker and binding pocket and have a lower molecular weight (i.e., ranging from 300 to 600 Da) than PROTAC (i.e., ranging from 700 to 1000 Da) [212].

### 6.3. Hydrophobic Tagging

Intracellular unfolded or misfolded proteins expose structurally modified hydrophobic amino acid residues or patches on their surface. These hydrophobic regions of partially denatured proteins can be recognized by the highly conserved and ubiquitous heat shock protein 70 (HSP70) chaperone. Subsequently, the co-chaperone E3 ligase C-terminus of Hsp70 interacting protein (CHIP) is recruited to induce the ubiquitination of the misfolded protein. As a result, misfolded proteins with exposed hydrophobic regions undergo quality control and degradation via the proteasome. Hydrophobic tagging (HyT) technology is a bifunctional molecule that induces the proteasomal degradation of POIs by mimicking misfolded proteins. HyT consists of a structure in which a hydrophobic moiety—e.g., an adamantyl group or Boc3Arg—and a selective ligand for a specific POI are connected by a short linker. Boc3Arg has been shown to degrade POIs by binding to the 20S proteasome involved in ATP and ubiquitin-independent degradation. However, the applications of this method are limited because it is known to inhibit Mammalian Target of Rapamycin Complex 1 signaling [213].

## 7. Conclusions and Future Perspectives

In this review, we summarized the basic mechanisms of proteasome-mediated degradation, its substrates, and various modalities of targeted degradation mediated by the proteasome. Decades of research on ubiquitin and proteasome have significantly broadened our understanding of the degradation mechanism and have been instrumental in elucidating signaling processes resulting from substrate degradation. Furthermore, the ongoing development of targeted degradation modalities and proteasome inhibitors is expected to position them as major therapeutic strategies and promising new therapeutic targets in the future.

For easier comprehension, we divided the degradation processes into those mediated by the 26S proteasome and the 20S proteasome. However, it is important to note that these two processes are not mutually exclusive; they are complementary to each other [140]. The 26S proteasome is not solely responsible for degrading ubiquitinated proteins, and the 20S proteasome does not exclusively target IDPs or oxidized proteins. Some studies have shown that certain proteins, such as ODC, can be degraded by the 26S proteasome without undergoing ubiquitination [67,214]. Additionally, ubiquitin-tagged proteins can also be degraded by the 20S proteasome [139]. Therefore, ubiquitin-dependent degradation via the 26S proteasome is widely recognized as the most common catalytic mechanism. However, simultaneous research on degradation through the 20S proteasome is also expected to continue steadily increasing.

Furthermore, the discovery of novel proteasome regulatory proteins is expected to greatly contribute to our understanding of the proteasome-mediated degradation process. Moreover, with the increasing research on degradation signaling pathways such as degron recognition and ubiquitin-like protein conjugation, there is a growing need for broad interest and extensive research in the field of the proteasome [26,215,216].

## Figures and Tables

**Table 1:** A list of substrate proteins degraded via 26S or 20S proteasome.

Proteasome	Ubiquitin	E3 Ligase	Substrate	Mediator	Disease	References
26S	dependent	MDM2	p53	RNF31	Cancer	[46,51]
			HIF1α		Cancer	[47]
		Siah2	ASPP2		Cancer	[52]
			PHD3		Cancer	[53]
			HIPK2		Cancer	[49]
			CHK2		Cancer	[54]
			HDAC3		Neurodegenerative disease	[55]
		Cullin3	HIF1α	RhoBTB3	Cancer	[56]
			ULK1	KLHL20	Cancer, Diabetes	[57]
		Roquin2	ASK1		Cancer	[50]
		VHL	HIF1α		Cancer	[58]
		KEAP1	IKKβ		Cancer	[59]
		KPC1	p105		Cancer	[60]
		CHIP	sGC		Cardiovascular disease	[61]
		Smurf1	PIPKIγ		Cancer	[62]
		CRL4	GRK2	Gβ2	Cardiovascular disease	[63]
		WWP1	KLF5		Cancer	[64]
		Itch	Bid		Cancer	[65]
		COP1	MTA1		Cancer	[66]
	independent		ODC	Antizyme1	Cancer	[67]
			TS		Cancer	[68]
20S	independent		p53		Cancer	[69]
			p53	Isg15	Cancer	[70]
			p35		Neurodegenerative disease	[71]
			p21	14-3-3τ	Cancer	[72]
			tau		Neurodegenerative disease	[73]
			α-synuclein		Neurodegenerative disease	[74]
			c-Myc	Antizyme2	Cancer	[75]
			IκBα		Cancer	[76]
			Aurora-A	AURKAIP1 Antizyme1	Cancer	[77,78]
			p130	pp71	Cancer	[79]
			Rb	MDM2	Cancer	[80]
			SE			[81]

**Table 2:** A list of proteasome regulatory proteins.

Proteasome Regulation	Family	Protein
Activation		11S
		PA200
		NRF1
		ZFAND5
		Tankyrase
Inhibition	CCRs ^1^	DJ-1
	CCRs	NQO1
		PI31
		c-Abl
		Bassoon
	CCRs	CBR3
	CCRs	KRas
	CCRs	RhoA

^1^ CCRs: catalytic core regulators.

**Table 3:** Comparison of different various targeted degradation modalities.

	Protac	Sniper	Molecular Glue	Hydrophobic Tagging
Degradation system	Ubiquitin–proteasome			
Active site in POIs	Not required			
Linker	Yes	Yes	No	Yes
Molecular weight	700–1000 Da	700–1000 Da	<500 Da	<500 Da
Feature	bivalent	Bivalent	monovalent	monovalent
Advantages	Entire elimination of pathogenic proteins			
	High selectivity			
	Low doses			
	Recycle			
Disadvantages	Low tissue penetration			
	Limited of E3 ligases			
	Lack of clinical data			
	Hook effect			
	Essential of target ubiquitination			
Targets	AR ^1^	AR	IKZF2 ^2^	AR
	ER ^3^	BRD4 ^4^		ALK ^5^
	BRD4			Tau
	BTK ^6^			SRC-1 ^7^
	Tau			Akt3 ^8^

^1^ AR: androgen receptor, ^2^ IKZF2: IKAROS family zinc finger 2, ^3^ ER: estrogen receptor, ^4^ BRD4: bromodomain containing 4, ^5^ ALK: anaplastic lymphoma kinase, ^6^ BTK: Bruton’s tyrosine kinase, ^7^ SRC-1: steroid receptor coactivator 1, ^8^ Akt3: v-akt murine thymoma viral oncogene homolog 3.

**Table 4:** Comparison of different various therapeutic modalities.

	PROTAC	SMI ^1^	Antibody	siRNA
Molecular weight	~1 kDa	<0.5 kDa	>150 kDa	5–15 kDa
Targeting intracellular proteins	Yes	Yes	No	Yes
Active site of target proteins	No required	Required	Required	No required
Elimination of target proteins	Yes	No	No	Yes
Tissue penetration	Low	High	Low	Low
Oral administration	Yes	Yes	No	No
Selectivity	High	Low	High	High
Doses	Low	High	High	Low
Stability	High	High	Low	Low

^1^ SMI: small molecular inhibitor.

## Data Availability

Not applicable.
